# Poster Session II - A303 VITAMIN D_3_ SUPPLEMENTATION AND ADHERENCE AMONG INFLAMMATORY BOWEL DISEASE PATIENTS

**DOI:** 10.1093/jcag/gwaf042.303

**Published:** 2026-02-13

**Authors:** S Hess, J Gregor, Y Zendo, E White, V Johnson, R MacMillan, T Ponich

**Affiliations:** London Health Sciences Centre, London, ON, Canada; London Health Sciences Centre, London, ON, Canada; London Health Sciences Centre, London, ON, Canada; London Health Sciences Centre, London, ON, Canada; London Health Sciences Centre, London, ON, Canada; Western University, London, ON, Canada; London Health Sciences Centre, London, ON, Canada

## Abstract

**Background:**

IBD is associated with reduced Vitamin D (VitD) levels and chronic inflammation. VitD supplementation has been linked to reduced relapse and improved patient quality of life (QoL). Adherence to medical management is a widespread concern in health care. We provided VitD supplementation to patients with IBD to assess adherence to medication with the ASK-12 (Adherence Starts with Knowledge 12-item) questionnaire and pill counting, and to measure a benefit to serum 25-hydroxy VitD levels.

**Aims:**

To characterize the IBD population, assess adherence to VitD Supplementation and outcome of supplementation on VitD levels and QoL.

**Methods:**

Patients suffering from Crohn’s Disease (CD) or Ulcerative Colitis (UC) under the care of LHSC Gastroenterology were recruited into this prospective cohort study. Participants were instructed to take one 1,000 IU VitD supplement daily and attend follow-up visits at 6 months (6MO) and 1 year (12MO). At each visit, patients provided blood samples for VitD levels, returned their supplement bottles for pill counts and completed two standardized questionnaires. Medication adherence was assessed using the ASK-12 questionnaire and health-related QoL was evaluated using the EQ-5D-5L questionnaire. Disease severity was measured using the Crohn’s Disease Activity Index (CDAI) or Mayo Score in UC. Patients were considered adherent if they took 80% or more of their supplements. ASK-12 cut-off score for adherence is accepted as 26.

**Results:**

131 patients participated in the study. 123 VitD results were available at Baseline; 102 at 6MO and 57 at 12MO, indicating the challenges of prospective research even with such simple design. There was a significant improvement in VitD levels from 68 ± 25 nmol/L at baseline to 79±26 at 6MO (p < 0.05) and a further slight, but not significant, increase to 83±22 at 12MO. The change in VitD levels did not depend on Type (CD vs UC) or Severity of Disease. Slightly more than half (55%) of patients returned pill counts at 6MO, but less than 30% returned pill counts at 12MO; this data was not analysed. ASK-12 responses were higher: 70% at 6MO but only 45% at 12MO. ASK-12 adherence values did not change across the study period (23±6 at each of baseline, 6MO and 12MO), QoL at Baseline (73±19) was not different from 6MO (77±14) but was significantly different at 12MO (82 ± 13).

**Conclusions:**

VitD levels improved across 12months of VitD supplementation in both UC and CD across all disease severities. VitD increased significantly in Females from baseline to 6MO and remained the same at 12MO. In Males the increase in VitD was not significant at 6MO but was significant at 12MO. QoL also increased over 12months.

**Funding Agencies:**

None

